# Effects of Grip Style and Contact Point on Force Production in a Tennis Forehand Groundstroke

**DOI:** 10.7759/cureus.74019

**Published:** 2024-11-19

**Authors:** Stephan Esser, Walter C Taylor, Raphael A O Bertasi, Livia Nishi, Michael G Heckman, Andre Abadin, Rock Vomer, George G. A Pujalte

**Affiliations:** 1 Department of Orthopedics, Southeast Orthopedic Specialists, Jacksonville, USA; 2 Department of Family Medicine, Mayo Clinic, Jacksonville, USA; 3 Internal Medicine, Mount Sinai Morningside West, New York, USA; 4 Department of Biostatistics, Mayo Clinic, Jacksonville, USA; 5 Orthopaedic Surgery, Duke University School of Medicine, Durham, USA

**Keywords:** forehand grip, sports, sports activities, sports biomechanics, tennis, tennis medicine

## Abstract

Modern tennis forehand grip style and ball contact points have evolved to enhance power and topspin. Different grip styles and ball contacts are recommended; however, little data are available to support one over another. Through a quantitative experimental study, we sought to determine which grip style and ball contact point produced the greatest forces at static contact. The continental, eastern, and semi-western forehand groundstroke grips of 100 volunteers were evaluated. Participants stood in three defined positions and applied maximum force against a dynamometer with each grip style. The associations of force produced at contact with the grip style, age, sex, tennis experience, and handedness were evaluated using univariate and multivariate linear mixed-effects regression models separately for early, mid, and late contact, including a random effect for each participant. Force production was significantly greater with eastern grip at early, mid, and late contact than with the other two grips (p < 0.001). Force was greater at early contact with semi-western grip than with continental. Force was also significantly greater for men at early, mid, and late contact (p < 0.001). Participants using an eastern grip were able to generate more force compared to those using a continental or semi-western grip, regardless of the ball contact point. Tennis players currently using other forehand grip styles who want to maximize power production may consider transitioning to an eastern forehand grip. The results of this study may improve tennis instruction and training for beginning and novice players who lack force generation.

## Introduction

Tennis is an asymmetric upper extremity sport that uses a racquet as a terminal extension of the arm. Contact between the racquet and the ball produces varied amounts of force, spin, angle, and loft. When contact occurs on the dominant side of a player’s body while standing near the baseline and after the ball has bounced, it is defined as a forehand groundstroke.

The forehand groundstroke involves a balance of power, control, and placement derived from the dominant arm, trunk, and legs. Power can be increased through various methods, including off-court strengthening, improved swing efficiency, racquet and string variables, and, most foundationally, modifications in technique that alter the ball-to-racquet contact [1].

Advancements in sports biomechanics have played a key role in understanding the mechanics of a tennis swing [2]. This plethora of information can help players improve their skills, limit injuries, and build a framework to practice their mastery. Stance positioning, muscle activation in a forehand stroke, and loading during a serve are a few variables that have been thoroughly researched over recent decades [2,3]. In addition, the kinetics of a tennis swing have been studied through analysis of the upper and lower extremities kinetic chain. Busuttil et al. [4] conducted a kinematic analysis on the double-handed backhand stroke with various grip positions. Interestingly, in studies on the biomechanics of tennis, there is a lack of research on power generation from a tennis swing with relation to grip style for a forehand stroke.

At the heart of the forehand groundstroke technique is the racquet grip style used. There are four primary grips recognized by the United States Professional Tennis Association [1]. These are the continental, eastern, semi-western, and western grips. Each grip alters the ideal contact point, power and spin production, and risk of injury [5]. The successful quest for increased power and spin production is reflected in the widespread transition in elite players away from the classic continental or universal grip style to the eastern and semi-western forehand grips [6]. The mechanics of an eastern and semi-western forehand grip facilitate an earlier contact point when greater shoulder and trunk rotation occurs [7]. In addition, these more modern grips bring the base of the index finger and the heel of the hand more completely behind the racquet strings [5].

Although there has been a transition to the use of these more modern grip styles for the forehand groundstroke, little scientific evidence exists to support this transition. Existing studies have focused mainly on grip size and grip pressure with the forehand stroke [8-15]. Our study was designed to investigate how altering grip type and contact point affects the static force produced at ball contact and to provide objective evidence for players and teaching professionals who seek to maximize force production. Our hypothesis is that there will be greater force production with the eastern and western grips compared to the continental grip, explaining why most professional tennis players use these modern grip styles.

## Materials and methods

In our quantitative experimental study, a total of 100 staff recruited from a regional hospital were included in this prospective study. All participants provided oral consent and volunteered to participate in the study in accordance with institutional review board approval. The participant mean (range) age was 41 (18-68) years, and the majority were women (71 (71.0%)). Only eight (8.0%) participants considered themselves tennis players and most were right-handed (92 (92.0%)).

**Figure 1:**
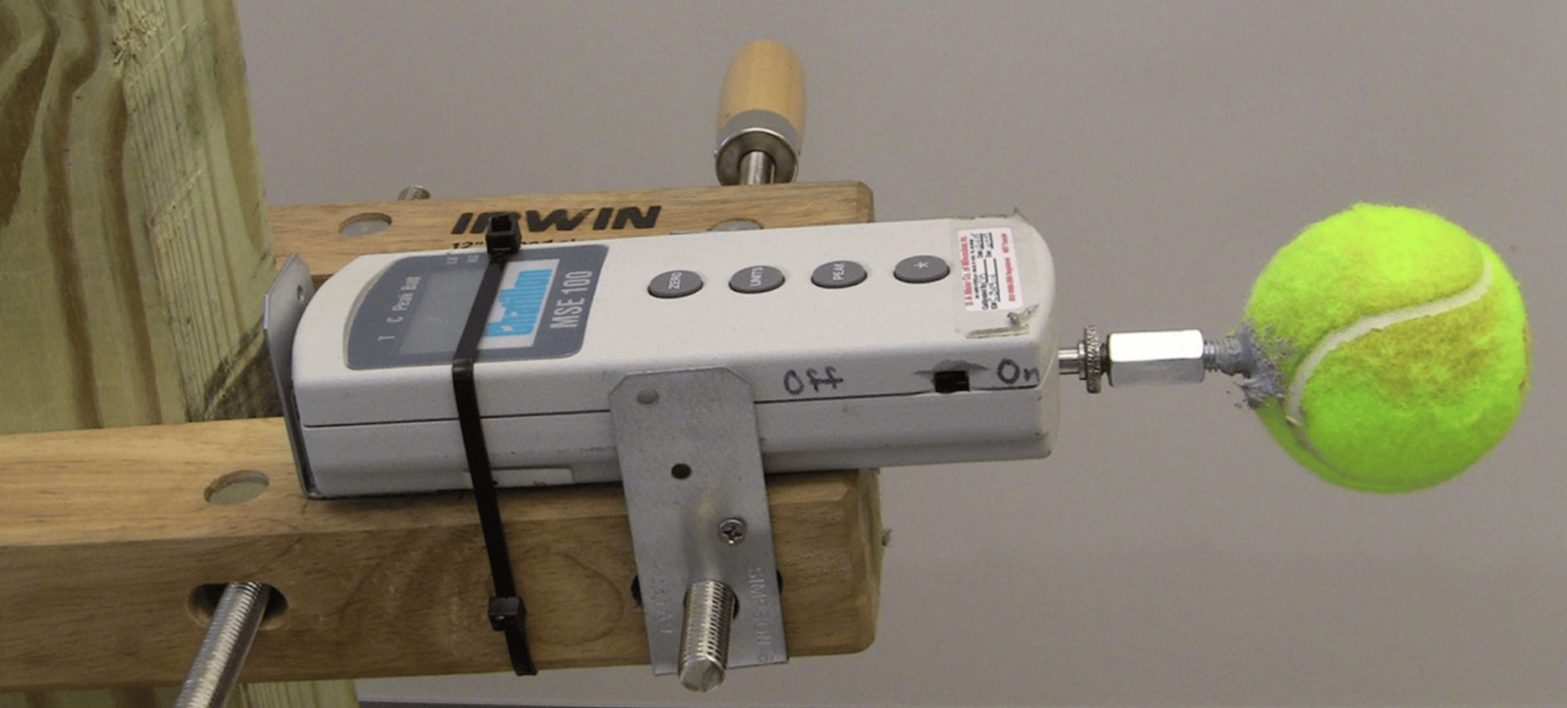
Dynamometer setup. The lag bolt and ball component were attached with a coupling to the mounting shaft of the dynamometer.

Static force at ball contact was measured using a Chatillon MSE100 Series dynamometer (AMETEK, Inc.). The device was mounted on a wooden frame affixed with screws and plates to a stationary metal storage unit, an adaptation of the design used by Ohguni et al. [14]. A standard regulation tennis ball was penetrated on one side with a sharp blade, and a 3/8×4 in steel lag bolt was placed, head first, inside. Then, a concrete binder was sprayed inside and allowed to harden. After curing, the ball was solid and without measurable compressibility. The lag bolt and ball component were then attached with a coupling to the mounting shaft of the dynamometer (Figure 1).

**Figure 2:**
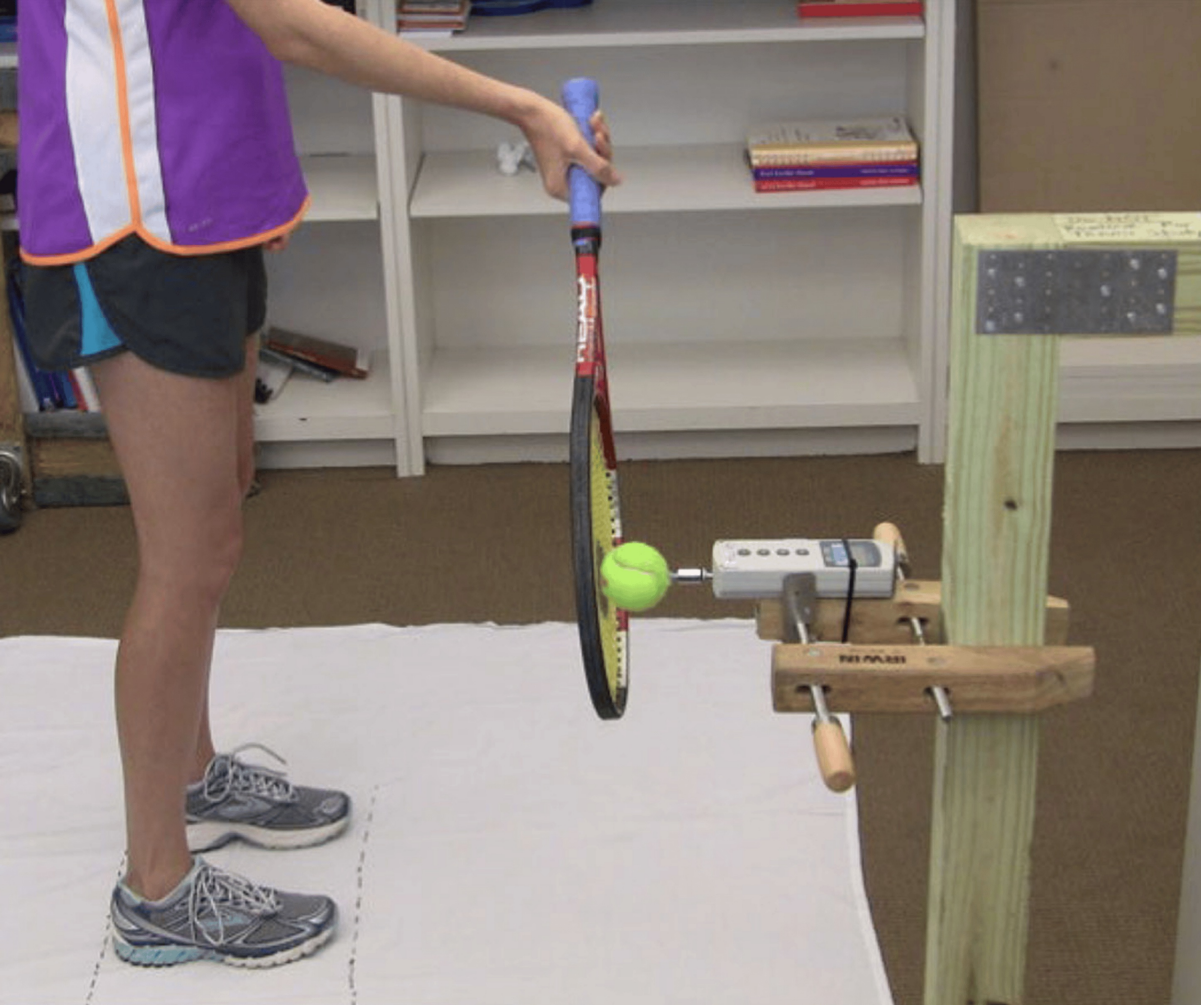
Dynamometer height adjustment. The height of the dynamometer was adjusted for each participant to be equal to the visual midpoint between the knee and the greater trochanter.

The height of the dynamometer was adjusted for each participant to be equal to the visual midpoint between the knee and the greater trochanter (Figure 2). Three separate locations for ball contact were then drawn on a movable mat so that all participants stood at the same distance from the ball and dynamometer interface. These points were defined as early (30 cm in front of the ipsilateral foot), mid (in the center of the body), and late (30 cm behind the contralateral foot). All participants stood in an open stance posture for all measurements and used a Prestige Midplus racquet (HEAD) strung with a 16-gauge head performance string at 24.9 kg of tension.

**Figure 3:**
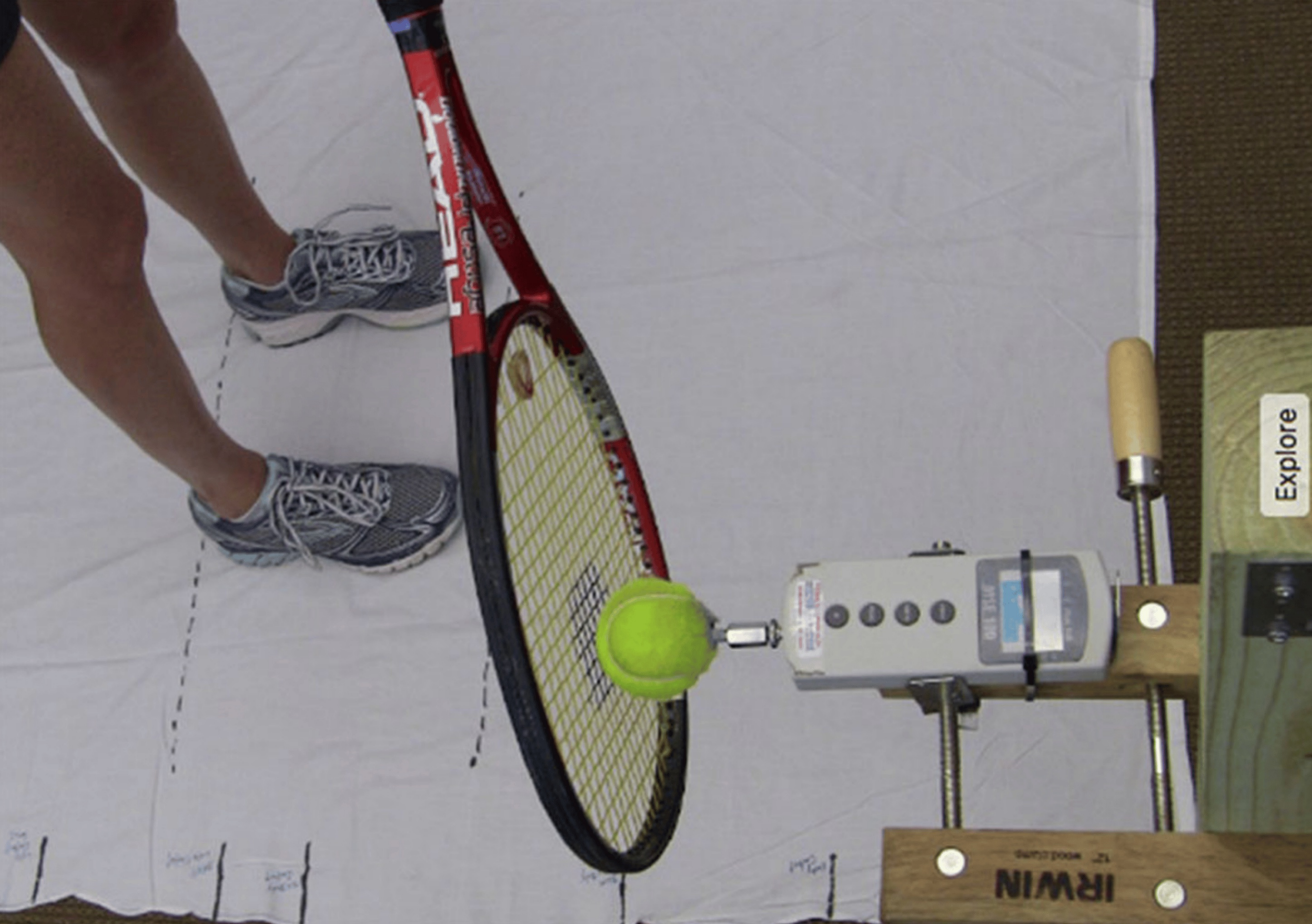
Racket stencil. A round stencil was painted on the center of the strings and matched up with the ball dynamometer interface for each test swing.

We used the same racquet and thus the same handle size for all participants to create an environment similar to a novice tennis player picking up a random racquet and using it. In our study, each participant also acted as their own relative control, and their force production was compared to themselves for each contact point, which eliminated grip size as a potential variable for force production. Postural control was obtained by having the participants stand on a specific mat with the same distance between the feet and distance to the ball and by altering the height of contact. A round stencil was painted on the center of the strings and matched up with the ball dynamometer interface for each test (Figure 3).

**Figure 4:**
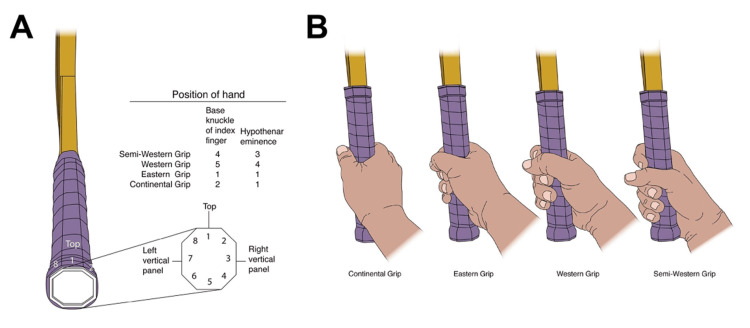
Grip types. A, Grip type is identified by where the second digit contacts the racket grip. B, Continental, eastern, and semiwestern grip types were used in this study. Credit: Mayo Clinic Art Department

**Figure 5:**
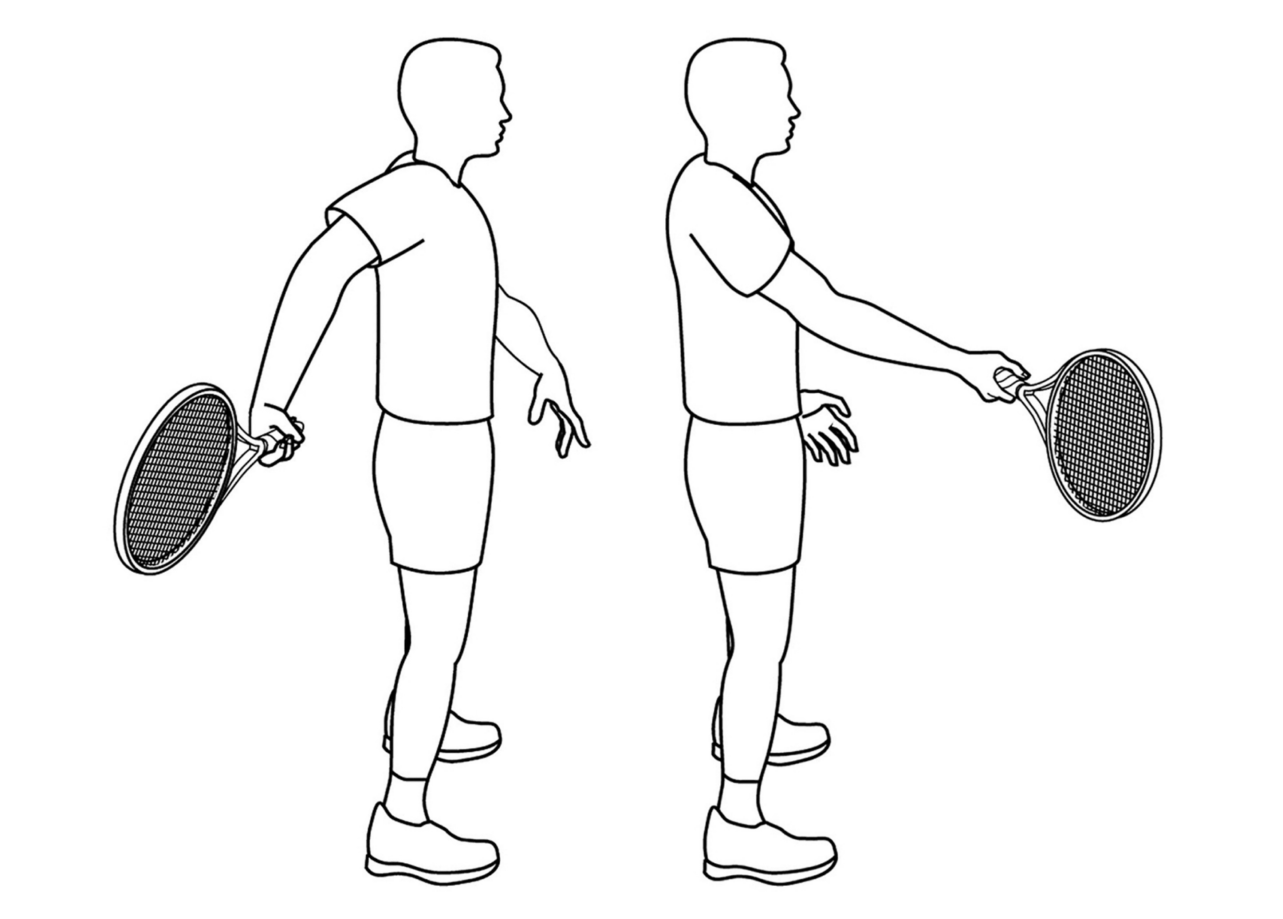
Force assessment positions. Maximal force produced at contact was measured for each of the contact locations at early to late swing. Credit: Mayo Clinic Art Department

The primary investigator demonstrated the technique, including grip style, posture, and maximal effort for each participant. The participant was then allowed one trial effort, after which all the following values were measured. The participants were asked to maintain maximal pressure for two seconds against the ball and dynamometer through the racquet without rotating their shoulders, trunk, or hips. Two seconds was selected as a measurable, moment, adequate for the participant to fire muscles, squeeze the racquet, and press against the dynamometer without being too long to lead to fatigue and without being too short to limit getting an accurate measurement. The maximal force produced at contact was measured for each of the three grip styles (i.e., continental, eastern, western, and semi-western) (Figures 4, 5) at each of the three contact locations (i.e., early, mid, and late) to yield nine different grip style and contact location scenarios. The force at each of the nine scenarios was measured twice, and the mean of the resulting two values was used in the analysis. To reduce the effect of fatigue on the findings, we ensured that the first 50 participants performed testing with early contact, followed by mid contact and then late contact. The next 50 performed testing in the opposite, with late, mid, and then early contact.

Continuous variables were summarized with the sample mean, SD, and range. Categorical variables were summarized with numbers and percentages. The associations of force produced at contact with the grip style, age, sex, tennis experience, and handedness were evaluated using univariate and multivariate linear mixed-effects regression models separately for early, mid, and late contact, including a random effect for each participant. Regression coefficients and 95% Cis were estimated. Multivariate models were adjusted for age, sex, grip style, whether the participant considered themselves’ a tennis player, and handedness. Multivariate analysis was not performed when evaluating the association between grip style and force at early contact, as the force was measured for each grip style for each study participant, which, with the random effect for the participant, included in the model, makes confounding by other variables of little concern. P values of 0.050 or less were considered statistically significant. All statistical analyses were performed using SAS, version 9.2 (SAS Institute Inc., USA) and R statistical software, version 2.14.0 (R Foundation for Statistical Computing, Vienna, Austria).

## Results

Data collected were force produced at contact with the grip style, age, sex, tennis experience, and handedness of the players. The associations of demographic and tennis characteristics with force at early, mid, and late contact are shown in Tables 1-3, respectively. For early contact (Table 1), the mean force was 1.37 Newtons (N) higher (95% CI, 0.08-0.20; p < 0.001) for the eastern grip and 0.69 N higher (95% CI, 0.00-0.12; p = 0.036) for the semi-western grip in comparison to the continental grip style. In addition, in comparison to the semi-western grip, the mean force at early contact was 0.69 N higher (95% CI, 0.01-0.13; p = 0.012) for the eastern grip.

**Table 1:** Association of force at early contact with demographic and tennis characteristics Abbreviation: N/A, not applicable a: Regression coefficients, 95% confidence intervals (CIs), and P-values result from linear mixed-effect regression models, including a random effect for each participant. Regression coefficients are interpreted as the mean difference in force at early contact in comparison with the reference category. Multivariable models were adjusted for grip style, age, sex, whether the participant considered themselves a tennis player, and handedness. b: Multivariable analysis was not performed when evaluating the association between grip style and force at early contact, as force at early contact was measured for each grip style in each study participant.

		Single-variable analysis		Multivariable analysis		
Variable	Mean ± SD (range) force at early contact	Regression coefficient (95% CI)^a^	P-value^a^	Regression coefficient (95% CI)^a^		P-value^a^
Grip style^b^		Test for overall difference: p < 0.001		N/A		
Continental	1.56 ± 0.76 (0.30-3.80)	Reference (0.00)	N/A	N/A	N/A	
Eastern	1.69 ± 0.84 (0.23-4.50)	0.14 (0.08-0.20)	<0.001	N/A	N/A	
Semi-western	1.62 ± 0.70 (0.25-3.60)	0.06 (0.00-0.12)	0.04	N/A	N/A	
Age, y		Test for overall difference: p = 0.53		Test for overall difference: p = 0.34		
≤30.0	1.48 ± 0.75 (0.23-4.15)	Reference (0.00)	N/A	Reference (0.00)		N/A
30.1-40.0	1.80 ± 0.85 (0.65-4.50)	0.31 (−0.11-0.74)	0.15	0.29 (−0.03-0.61)		0.07
40.1-55.0	1.66 ± 0.71 (0.43-3.73)	0.18 (−0.20-0.55)	0.35	0.16 (−0.13-0.44)		0.28
>55.0	1.61 ± 0.78 (0.45-3.80)	0.13 (−0.31-0.57)	0.56	0.15 (−0.18-0.47)		0.38
Sex						
Female	1.30 ± 0.41 (0.23-2.85)	Reference (0.00)	N/A	Reference (0.00)		N/A
Male	2.41 ± 0.86 (0.58-4.50)	1.11 (0.87-1.35)	<0.001	1.12 (0.88-1.37)		<0.001
Tennis player						
No	1.61 ± 0.76 (0.23-4.15)	Reference (0.00)	N/A	Reference (0.00)		N/A
Yes	1.78 ± 0.91 (0.58-4.50)	0.17 (−0.37-0.72)	0.53	−0.18 (−0.61-0.25)		0.41
Handedness						
Left	1.37 ± 0.43 (0.60-2.23)	Reference (0.00)	N/A	Reference (0.00)		N/A
Right	1.65 ± 0.79 (0.23-4.50)	0.28 (−0.27-0.82)	0.32	0.13 (−0.28-0.54)		0.54

For mid contact (Table 2), in comparison to the continental grip style, the mean force was 0.78 N higher for the eastern grip (95% CI, 0.03-0.13; p = 0.002), but it was not significantly different for the semi-western grip (p = 0.550). The mean force at mid contact was 0.88 N higher (95% CI, 0.04-0.14; p < 0.001) for the eastern grip style than for the semi-western grip.

**Table 2:** Association of force at mid contact with demographic and tennis characteristics Abbreviation: N/A, not applicable a: Regression coefficients, 95% CIs, and P values result from linear mixed-effects regression models, including a random effect for each participant. Regression coefficients are interpreted as the mean difference in force at mid contact in comparison with the reference category. Multivariable models were adjusted for grip style, age, sex, whether the participant considered themselves a tennis player, and handedness. b: Multivariable analysis was not performed when evaluating the association between grip style and force at early contact, as the force at mid contact was measured for each grip style in each study participant.

		Single-variable analysis		Multivariable analysis	
Variable	Mean ± SD (range) force at mid contact	Regression coefficient (95% CI)^a^	P-value^a^	Regression coefficient (95% CI)^a^	P-value^a^
Grip style^b^		Test for overall difference: p=0.01		N/A	
Continental	1.46 ± 0.65 (0.40-3.85)	Reference (0.00)	N/A	N/A	N/A
Eastern	1.54 ± 0.69 (0.35-3.60)	0.08 (0.03-0.13)	0.002	N/A	N/A
Semi-western	1.45 ± 0.65 (0.28-3.53)	−0.01 (−0.06-0.03)	0.550	N/A	N/A
Age, y		Test for overall difference: p=0.40		Test for overall difference: p=0.200	
≤30.0	1.32 ± 0.61 (0.35-2.95)	Reference (0.00)	N/A	Reference (0.00)	N/A
30.1-40.0	1.54 ± 0.70 (0.63-3.60)	0.22 (−0.14-0.59)	0.230	0.21 (−0.07-0.50)	0.140
40.1-55.0	1.58 ± 0.60 (0.28-3.53)	0.26 (−0.06-0.58)	0.110	0.26 (−0.00-0.51)	0.050
>55.0	1.55 ± 0.76 (0.58-3.85)	0.23 (−0.15-0.61)	0.240	0.24 (−0.05-0.53)	0.110
Sex					
Female	1.22 ± 0.39 (0.28-2.28)	Reference (0.00)	N/A	Reference (0.00)	N/A
Male	2.14 ± 0.74 (0.65-3.85)	0.92 (0.70-1.13)	<0.001	0.92 (0.70-1.14)	<0.001
Tennis player					
No	1.47 ± 0.65 (0.28-3.85)	Reference (0.00)	N/A	Reference (0.00)	N/A
Yes	1.68 ± 0.77 (0.65-3.60)	0.21 (−0.26-0.68)	0.380	−0.04 (−0.42-0.34)	0.830
Handedness					
Left	1.36 ± 0.38 (0.68-2.03)	Reference (0.00)	N/A	Reference (0.00)	N/A
Right	1.50 ± 0.68 (0.28-3.85)	0.14 (−0.33-0.61)	0.560	0.03 (−0.34-0.39)	0.880

At late contact (Table 3), in comparison to the continental grip style, the mean force was 1.27 N higher (95% CI, 0.08-0.17; p < 0.001) for the eastern grip, but it was not significantly different for the semi-western grip (p = 0.520). In comparison to the semi-western grip style, the mean force at late contact was 1.37 N higher (95% CI, 0.10-0.19; p < 0.001) for the eastern grip.

**Table 3:** Association of force at late contact with demographic and tennis characteristics Abbreviation: N/A, not applicable a: Regression coefficients, 95% confidence intervals, and P values result from linear mixed-effects regression models, including a random effect for each participant. Regression coefficients are interpreted as the mean difference in force at mid contact in comparison with the reference category. Multivariable models were adjusted for grip style, age, sex, whether the participant considered themselves a tennis player, and handedness. b: Multivariable analysis was not performed when evaluating the association between grip style and force at early contact, as force at late contact was measured for each grip style in each study participant.

		Single-variable analysis		Multivariable analysis		
Variable	Mean ± SD (range) force at late contact	Regression coefficient (95% CI)^a^	P-value^a^	Regression coefficient (95% CI)^a^	P-value^a^	
Grip style^b^		Test for overall difference: p<0.001		N/A		
Continental	1.25 ± 0.56 (0.20-3.50)	Reference (0.00)	N/A	N/A		N/A
Eastern	1.37 ± 0.63 (0.25-3.78)	0.13 (0.08-0.17)	<0.001	N/A		N/A
Semi-western	1.23 ± 0.57 (0.25-3.00)	−0.01 (−0.06-0.03)	0.520	N/A		N/A
Age, y		Test for overall difference: P=0.510		Test for overall difference: p=0.280		
≤30.0	1.16 ± 0.53 (0.25-3.00)	Reference (0.00)	N/A	Reference (0.00)		N/A
30.1-40.0	1.27 ± 0.60 (0.48-2.83)	0.12 (−0.21-0.44)	0.480	0.11 (−0.16-0.38)		0.410
40.1-55.0	1.38 ± 0.54 (0.20-2.90)	0.23 (−0.06-0.51)	0.120	0.23 (−0.01-0.47)		0.060
>55.0	1.33 ± 0.71 (0.40-3.78)	0.18 (−0.16-0.51)	0.300	0.19 (−0.09-0.47)		0.180
Sex						
Female	1.07 ± 0.37 (0.20-2.05)	Reference (0.00)	N/A	Reference (0.00)		N/A
Male	1.80 ± 0.69 (0.58-3.78)	0.72 (0.52-0.93)	<0.001	0.72 (0.51-0.93)		<0.001
Tennis player						
No	1.27 ± 0.58 (0.20-3.78)	Reference (0.00)	N/A	Reference (0.00)		N/A
Yes	1.47 ± 0.67 (0.58-2.83)	0.20 (−0.22-0.61)	0.350	0.01 (−0.36-0.37)		0.980
Handedness						
Left	1.14 ± 0.37 (0.58-2.00)	Reference (0.00)	N/A	Reference (0.00)		N/A
Right	1.30 ± 0.60 (0.20-3.78)	0.15 (−0.26-0.57)	0.47	0.06 (−0.29-0.41)		0.73

For early, mid, and late contact, the force was significantly higher for men than for women (all p < 0.001; Tables 1-3). There was no evidence of an association between force and age, whether or not the participants considered themselves tennis players, or handedness (all p ≥ 0.190; Tables 1-3).

**Figure 6:**
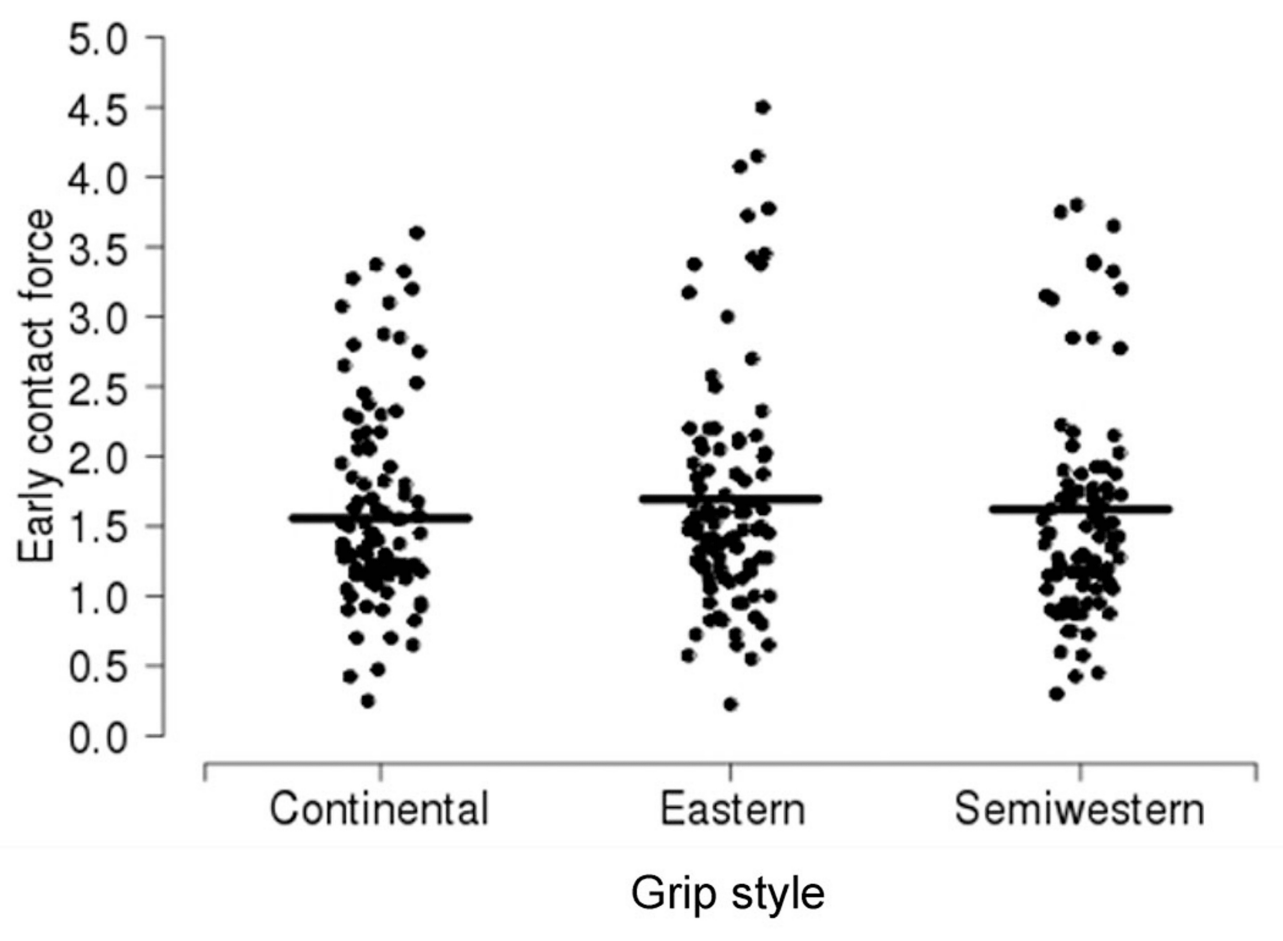
Early contact force according to grip style. The sample mean is indicated by a solid horizontal line.

**Figure 7:**
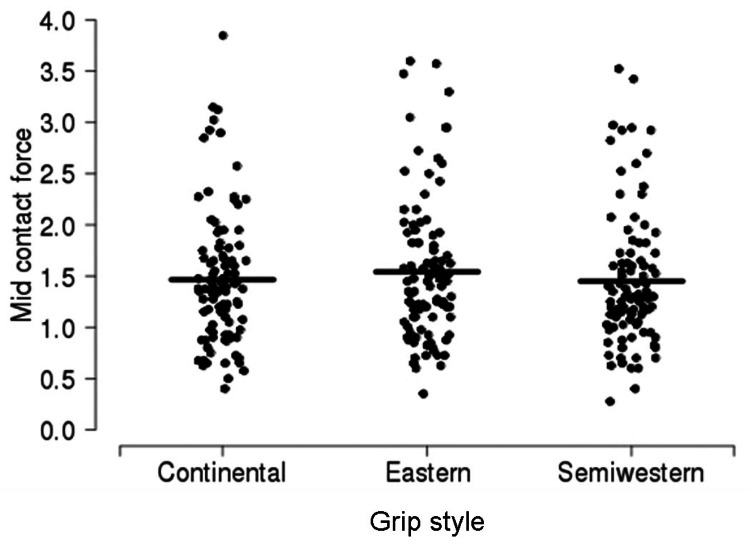
Mid contact force according to grip style. The sample mean is indicated by a solid horizontal line.

**Figure 8:**
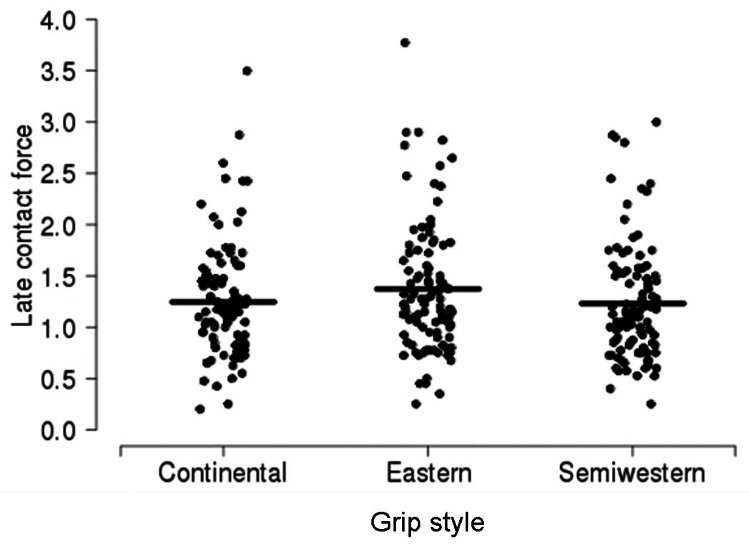
Late contact force according to grip style. The sample mean is indicated by a solid horizontal line.

A summary of force according to contact location is shown in Table 4, separately for each grip style (Figures 6-8). Overall, compared to early contact, the force was 1.37 N lower (95% CI, 0.10-0.18; p < 0.001) for mid contact and 0.34 units (95% CI, 0.30-0.38; p < 0.001) units lower for late contact. Compared to mid contact, the force was 1.96 N lower (95% CI, 0.16-0.24; p < 0.001) for late contact. There was no evidence of an interaction between grip style and contact location with regard to force (p = 0.320); these differences in force between contact locations were similar when each grip style was considered separately.

**Table 4:** Summary of force at each location of contact according to grip style

Grip style	Location of contact	Mean ± SD (range) force (N)
Continental	Early contact	15.30 ± 7.45 (2.94-37.27)
Continental	Mid contact	14.32 ± 6.37 (3.92-37.76)
Continental	Late contact	12.26 ± 5.49 (1.96-34.32)
Eastern	Early contact	16.57 ± 8.24 (2.26-44.13)
Eastern	Mid contact	15.10 ± 6.77 (3.43-35.31)
Eastern	Late contact	13.43 ± 6.18 (2.45-37.07)
Semi-western	Early contact	15.89 ± 6.86 (2.45-35.31)
Semi-western	Mid contact	14.22 ± 6.37 (2.75-34.62)
Semi-western	Late contact	12.06 ± 5.59 (2.45-29.42)

## Discussion

To the knowledge of the study investigators, this is the first study to compare the static force produced by three different forehand grip styles. This study’s investigators have elected to focus on continental, eastern, and semi-western grip styles as these account for the majority of grip styles used by beginner competitors [5]. We provide evidence that the force is significantly greater for the eastern grip style than for the continental and semi-western grips at early, mid, and late contact. The force is significantly greater at early contact for the semi-western grip style than for the continental grip. In addition, the measured force is significantly greater for men than for women at each contact location, and it is greatest at early contact, followed by mid contact and then late contact.

Ohguni et al. [14] used a similar method to measure the static force produced by multiple participants with the forehand stroke. However, the authors measured the effects of different forehand grip sizes, and all participants used only the semi-western grip with the same contact point [14]. Busuttil et al. [4] analyzed forces with different grip styles for double-handed backhand strokes, but their measurements were ball speed and revolutions and they did not examine forehand strokes. Similar to Ohguni et al. [14] and other studies [16,17], they found that male participants generated a greater force than female participants. Male participants with greater pincer grip strength and longer middle fingers achieve greater force with the largest grip size [14,18].

The forehand groundstroke grip style necessitates altering the contact point and stroke technique. The classic forehand tennis stroke uses a continental grip [5]. In this grip, the second metacarpal phalangeal joint of the dominant hand lays over the second bevel of the grip (Figure 4). As a result, the heel of the hand is directed more toward the ground. The ideal contact point with this grip style is in the center of the body or at the mid-location. Here, the racquet face is in a neutral position, and maximal power can be produced. However, when the grip is rolled forward so that the third (eastern) or fourth (semi-western) bevel lies beneath the second metacarpal phalangeal joint, the heel of the hand is located more behind the racquet handle. As a result, it is believed that a larger force vector is translated forward and into the ball. However, the eastern and semi-western grips place the racquet face into a more closed position, meaning the strings face toward the ground (Figure 5). Consequently, the ball must be struck earlier with regard to the body, so the racquet face will be more upright at contact. Thus, the ideal contact point for using eastern and semi-western grips is in front of the body (Figures 5-8). At this location, the hips and shoulders have rotated further, and more force from these larger muscle groups can be translated into the ball, rather than be used to pull the ball from behind the shoulders and guide it along the desired trajectory [1].

This study suggests that beginning tennis players may not be achieving optimal power with a continental grip. Anecdotally, players who use a continental grip use less shoulder and hip rotation, as well as more elbow and shoulder acceleration to create power. Using the smaller muscle groups to produce power is less efficient and may increase the risk of shoulder and elbow injury [3]. A study evaluating forehand grip style and the development of wrist injuries found that in nonprofessional players with wrist injuries, the western or semi-western grip was more associated with ulnar-sided injuries and the eastern grip with radial-sided injuries [5]. 

A limitation of this study was that most of the participants were not tennis players, which may have affected their ability to feel comfortable holding the racquet and, therefore, limited the force generation. However, this group would be similar to novice and recreational players. In addition, the same racquet, and thus grip size, was used by all participants with varying hand sizes, which might have affected their force production. Non-tennis players or those who were only moderately active were intentionally selected to reduce bias. If an athlete used a certain grip style regularly and believed it was best for them, it could be assumed that this preference would make the participant press harder when they used their favored grip. In addition, if a participant played tennis regularly, they would have had resultant hypertrophy of forearm musculature, enabling them to produce more force with their specific grip style. In truth, the study was intended to shed light on how an untrained individual, not a trained or an elite player, could produce force. Further studies need to investigate the relationship between grip styles and tennis performance (e.g., does the ability to produce static force affect the ability to stabilize racquet kinematics during off-center impact?). The ability of grip styles to help stabilize the racquet at contact needs to be assessed further. However, this is the first study to describe the amount of static force that can be produced by untrained individuals when they use three different forehand grips while pressing in three different locations in reference to the body. This is also the first study to capture data from a single reducible moment in time (i.e., the moment of contact). It is worthwhile to note the dynanometer used in the study was very accurate. The investigators feel that the observed differences were relevant with respect to the experimental setup. Another limitation is that force measurements were obtained in a static manner rather than with a dynamic normal tennis forehand groundstroke at ball contact.

Future studies need to examine if the velocity and angle of the ball are more heavily influenced by racquet movement than by the forces related to grip style. While the position order was changed between the first 50 participants and the second, there was no sequencing of grip styles. This could be done in future studies. A formal procedure for counterbalancing conditions could also be attempted. Finally, this study does not examine grip force but focuses on grip style only. Future studies are required to further elucidate the interplay of these factors.

## Conclusions

To our knowledge, this is the first study to compare the static force produced by the three forehand grip styles (i.e., continental, eastern, and semi-western) most often used by beginner tennis competitors. Forces generated were significantly greater for the eastern grip style compared to the continental and semi-western grips at early, mid, and late contact. Compared to early contact, forces were lower for the mid contact and late contact. There appeared to be no relationship between grip styles and contact locations with regard to force, indicating that the differences in force between contact locations were similar within each grip style when considered separately. Forces were significantly greater at early contact for the semi-western grip style compared to the continental grip. This study revealed that participants using an eastern grip were able to generate more force compared to those using a continental or semi-western grip, regardless of the ball contact point. As a practical application, tennis players currently using other forehand grip styles who want to maximize power production may consider transitioning to an eastern forehand grip. The results of this study may improve tennis instruction and training for beginning and novice players who lack force generation. While this study is not intended to be the final word in force production, it seeks to add to the dialogue on it. Further research is needed to determine the effects of players’ use of specific forehand grips on power production, accuracy, and risk of injury.
